# DNA Barcoding Identification of *Angelicae Sinensis* Radix and Its Adulterants Based on Internal Transcribed Spacer 2 Region and Secondary Structure Prediction

**DOI:** 10.3390/genes16111333

**Published:** 2025-11-05

**Authors:** Zifeng Chen, Qiman Zeng, Meihui Gong, Huimin Wu, Wenli Chen, Xinjun Xu

**Affiliations:** 1 School of Pharmaceutical Sciences, Sun Yat-Sen University, Guangzhou 510275, China; chenzf39@mail2.sysu.edu.cn (Z.C.); m458871895@163.com (Q.Z.); 2 Pharmacy Department, Fifth Affiliated Hospital, Sun Yat-Sen University, Zhuhai 519000, China; gongmh@mail.sysu.edu.cn (M.G.); wuhm23@mail.sysu.edu.cn (H.W.)

**Keywords:** *Angelicae Sinensis* Radix, *Ligusticopsis Pubescens* Radix, *Angelicae Pubescens* Radix, ITS2, DNA barcoding

## Abstract

**Background**: Morphological similarities among *Angelicae Sinensis* Radix and related species often lead to market substitution. This study develops a DNA barcoding method to authenticate these herbs and identify adulterants derived from *Angelica*, *Heracleum*, and *Peucedanum* genera. **Methods**: Phylogenetic analysis was conducted using MEGA 11.0 software with ITS2 sequences from *Angelicae Sinensis* Radix, *Ligusticopsis Pubescens* Radix, *Angelicae Pubescens* Radix, and their related species within the genera *Angelica*, *Peucedanum*, and *Heracleum*. Additionally, ITS2 secondary structures were predicted for the three herbs to provide supplementary evidence for identification. **Results:** The amplification success rate was 86.67%, and the interspecific genetic distance, ranging from 0.009~0.220, was significantly greater than the intraspecific genetic distance range from 0.000~0.062, indicating that the ITS2 sequence is suitable for differentiating three herbs and their related species. Additionally, their ITS2 secondary structures exhibited significant differences, which can also serve as a reliable criterion for their identification. **Conclusions:** This study not only validated the identification efficacy of ITS2 sequences and their secondary structures for the three herbs, but more importantly, enabled precise traceability of adulterants through the construction of a comprehensive phylogenetic framework.

## 1. Introduction

*Angelicae Sinensis* Radix (ASR) is a traditional Chinese medicinal herb, renowned as the “holy herb for blood-related disorders”, which contains angelica polysaccharides, organic acids, and volatile oils. It exhibits blood-tonifying, blood-activating, menstruation-regulating, and pain-relieving effects and is widely used in treating gynecological and cardiovascular diseases. However, due to morphological similarities, regional usage variations, and nomenclature inconsistencies, ASR is frequently confused with other species (such as *Angelicae Pubescens* Radix (APR) and *Ligusticopsis Pubescens* Radix(LPR)), which severely compromises drug quality and therapeutic efficacy. APR is renowned for its wind-dispelling, dampness-resolving, and pain-relieving properties, chiefly employed in the treatment of rheumatic arthralgia [1]. LPR is commonly applied in respiratory disorders [2]. Misidentification may lead to reduced efficacy or even adverse reactions, making accurate discrimination crucial. Traditional techniques for identifying traditional Chinese medicine include morphological, microscopic, spectroscopic, and chromatographic analyses [3]. Morphological identification and microscopic identification are susceptible to subjective factors, while spectroscopic and chromatographic identification may cause composition fluctuations due to environmental factors. In recent years, DNA barcoding technology has emerged as a powerful tool for species identification due to its high accuracy, reproducibility, and independence from developmental stages and environmental influences. The ITS2 sequence, characterized by its high variability and universality, has been widely adopted for plant species discrimination [4,5,6]. Moreover, ITS2 secondary structure analysis can further enhance identification precision by detecting compensatory mutations that are undetectable through sequence alignment. Accordingly, a growing number of studies are now integrating ITS2 sequences with their secondary structures for molecular identification of species [7,8,9].

While recent studies have employed DNA barcoding to distinguish *Angelica sinensis* (Oliv.) Diels from certain adulterants [10,11,12], their scope remains limited to specific substitution types. These investigations primarily focus on distinguishing a restricted number of common adulterants and lack comprehensive analysis of complex substitution patterns across multiple genera. However, commercial ASR exhibits polyphyletic adulteration, with contaminants deriving from both conspecific variants and intergeneric (such as *Peucedanum* and *Heracleum* genera) closely related species. The existing identification framework shows notable limitations in covering the diverse range of phylogenetically distant yet morphologically similar adulterants found in commercial markets.

To address this gap, this study develops an ITS2-based DNA barcoding system to authenticate ASR and its common substitutes. By constructing a comprehensive phylogenetic framework encompassing *Angelica*, *Peucedanum*, and *Heracleum* genera, we overcome previous limitations in adulterant coverage and establish a reliable method for genus-level traceability in herbal medicine quality control.

## 2. Materials and Methods

### 2.1. Materials

**Figure 1:**
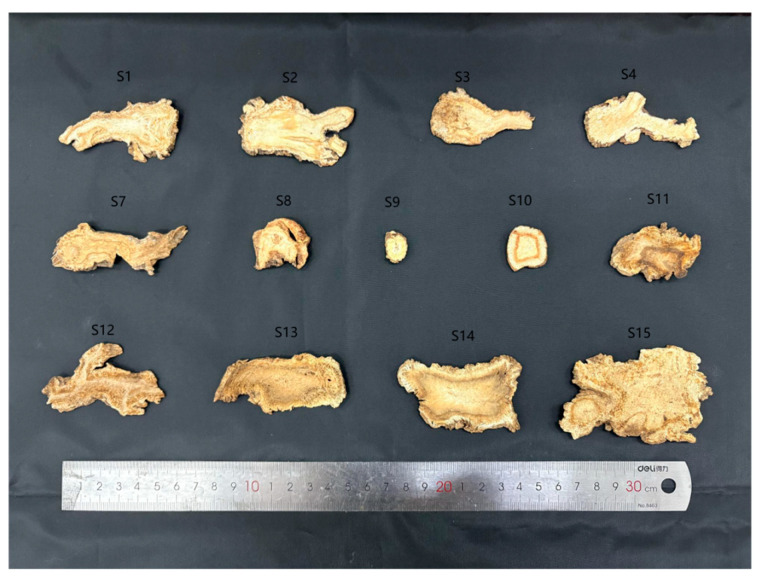
Slice diagram of *Angelicae Sinensis* Radix, *Ligusticopsis Pubescens* Radix, and *Angelica Pubescens* Radix.

**Figure 2:**
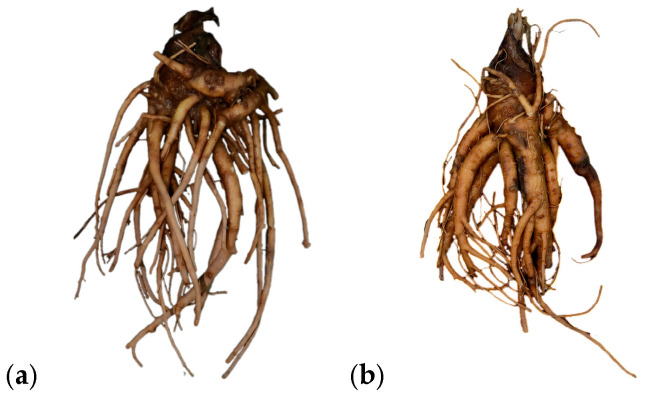
Appearance images of herbal materials S5 (**a**) and S6 (**b**) (S5 and S6 are whole-plant morphologies and are not sliced).

The primary instruments used in this study included the following: herb grinder (Ruian Baixin Pharmaceutical Machinery Co., Ltd., LG-04, Ruian, China), electronic balance (CPA225D, Sartorius Göttingen, Germany), thermostatic water bath (Yuhua Instrument Co., Ltd., DF101S, Gongyi, China), centrifuge (Eppendorf AG, Hamburg, 5417R, Germany), polymerase chain reaction amplification (Hangzhou Baiheng Technology Co., Ltd., Repure A, Hangzhou, China), and electrophoresis apparatus (BIO-RAD, PowerPac Basic, Hercules, CA, USA). Anhydrous MgCl_2_, NaOH, anhydrous ethanol, isoamyl alcohol, chloroform, and mercaptoethanol were of analytical grade and manufactured by Shanghai Acmec Biochemical Technology Co., Ltd. (Shanghai, China); TaqPCR Master Mix (×2, blue dye) was purchased from Shanghai Acmec Biochemical Technology Co., Ltd.; The TE buffer, TAE buffer, and agarose, were purchased from Sangon Biotech (Shanghai, China). Goldview was purchased from MYM Biological Technology Co., Ltd. (Beijing, China). HP Plant DNA mini kit was purchased from Omega Bio-tek (D2485-01, Norcross, GA, USA). ASR was purchased from different real estate areas in China; LPR was purchased from Anhui Province, Hebei Province, Shanxi Province, and Guangdong Province; and APR was purchased from Huoshan Mountain in Anhui Province, Jieyang City in Guangdong Province, and Gansu Province (Table S1, Figure 1 and Figure 2). The GenBank accession numbers for the ITS2 sequences from related species of ASR, LPR, and APR are presented in Table S2.

### 2.2. Methods

**Figure 3:**
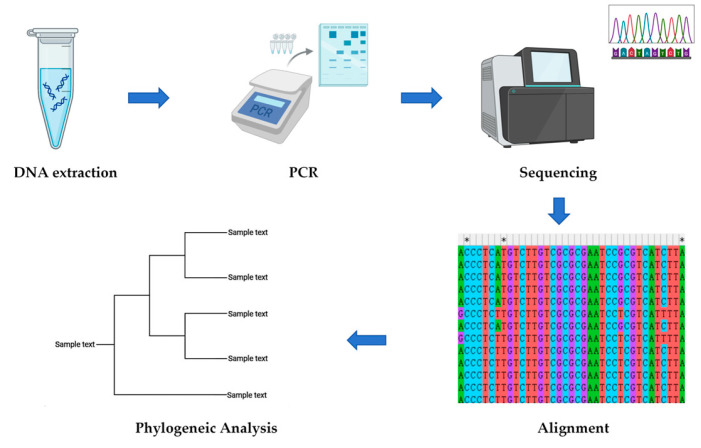
Workflow of the molecular authentication.

The general experimental workflow for the molecular authentication is illustrated in Figure 3. The DNA extraction procedure began by wiping fifteen samples with 75% ethanol for surface decontamination before grinding them into a fine powder. Approximately 40~60 mg of the powdered dry tissue was accurately weighed and transferred into 2 mL nuclease-free microcentrifuge tubes. Then, 600 μL of CSPL buffer was added to each tube, followed by vigorous vortexing to ensure complete mixing and the dispersion of any clumps. The samples were subsequently incubated at 65 °C for 30 min, with the tubes being inverted twice during this incubation period to promote uniform processing. Following incubation, 600 μL of chloroform/isoamyl alcohol (24:1) was added to each sample, which was again vortexed thoroughly to achieve a homogeneous mixture. The tubes were then centrifuged at 12,000 rpm for 10 min to separate the phases. After centrifugation, 300 μL of the aqueous phase was carefully transferred to a new microcentrifuge tube. To precipitate the DNA, 150 μL of CXD Buffer and 300 μL of 100% ethanol were added to the aqueous phase, with vortexing performed to obtain a uniform solution. The resulting mixture was loaded onto HinBind DNA Mini Columns inserted in 2 mL Collection Tubes and centrifuged at 12,000 rpm for 2 min. After discarding the filtrate, the column was washed twice with the provided wash buffer to ensure complete purification. For final elution, 50 μL elution buffer, pre-heated to 65 °C, was added to the column, which was then centrifuged at 12,000 rpm for 3 min. This elution step was repeated twice. The purified DNA was ultimately stored at −20 °C for long-term preservation. The extracted DNA was quantified using ultra-trace UV-Vis spectrophotometry. The A_260_/A_280_ ratio served as an indicator of purity, with a value between 1.80~2.00 considered acceptable for pure DNA. DNA barcodes were amplified by PCR using universal primers (S2F: 5′-ATG CGATACTTG GTGTGAAT-3′ and S3R: 5′-GACGCTTCTCCAGACTACAAT-3′). Each 25 μL reaction mixture contained 12.5 μL Taq PCR Master Mix, 1 μL Genomic DNA (about 10~20 ng), 1 μL of each 10 μM primer, 1 μL MgCl_2_ (20 mmol/L), and ddH_2_O 8.5 μL. The PCR conditions for amplification were as follows: initial denaturation at 94 °C for 5 min; 40 cycles of denaturation at 94 °C 30 s, annealing at 56 °C for 30 s, and extension at 72 °C for 45 s; a final extension at 72 °C for 10 min, and a final hold 4 °C. The PCR product was detected by electrophoresis on a 2% agarose gel. A measure of 25 μL of PCR product was loaded into the wells, and the electrophoresis was performed at 120 V for 40 min in 1× TAE buffer. The bands were visualized under ultraviolet light. Target bands were excised and sent to Sangon Biotech Co., Ltd. (Shanghai, China), for purification and sequencing. The obtained sequences were subjected to BLAST (2.17.0) similarity searches against the NCBI database. They were then annotated, trimmed, and their secondary structures were predicted using the website (http://its2.bioapps.biozentrum.uni-wuerzburg.de/, accessed on 20 October 2025) established by Schultz et al. [13,14]. The sequences were aligned using the ClustalW algorithm. Key alignment parameters included a gap opening penalty of 15.00 and a gap extension penalty of 6.66 for both pairwise and multiple alignment stages. The DNA weight matrix was set to IUB with a transition weight of 0.50. The delay divergent cutoff was set to 30%, and the use negative matrix and keep predefined gap options were disabled. Sequence divergence was quantified using pairwise genetic distances calculated under the Kimura 2-parameter (K2P) model. A neighbor-joining (NJ) phylogenetic tree was reconstructed based on this model, with gaps handled by complete deletion. The robustness of the tree topology was assessed by bootstrap analysis with 1000 replicates.

## 3. Results

### 3.1. Total DNA Extraction

The concentration and purity of the total DNA extraction are shown in Table 1. It is worth noting that although the A_260_/A_280_ ratios of some DNA samples were slightly higher than 2.0, which might be due to residual secondary metabolites or trace RNA in the medicinal plant roots, this had minimal impact on the PCR amplification of the ITS2 sequences. These samples were thus suitable for subsequent PCR reactions.

### 3.2. PCR Results and Sequence Characteristics

#### 3.2.1. PCR Results

Figures S1 and S2 show the PCR results. The amplification and sequencing success rate of the three traditional Chinese medincines was 86.67%. The DNA degradation of S7 samples was severe and failed to be amplified and other samples were successfully amplified. Furthermore, there were overlapping peaks in S5 sequencing. Based on our observations, we conclude that the failed amplification of sample S7 likely resulted from DNA degradation due to prolonged storage, while sample S5 exhibited characteristics consistent with potential contamination, so the groups of S7 and S5 were discarded.

#### 3.2.2. Sequence Characteristics

BLAST analysis against the NCBI database identified the successfully amplified sequences as follows; samples S1~S4, S6 and S9 were ASR, while S8 and S10 were LPR, and S11~S15 were APR. However, sample S9, which was originally labeled as LPR, was genetically identified as ASR. The ITS2 sequences of ASR, LPR, and APR were quite different, with a total of 178 differential sites. The sequence length and GC content of each sample are shown in Table 2. The ITS2 sequences for the closely related species of ASR, LPR, and APR exhibited a length range of 224~229 bp and a GC content of 52.89~58.67%. The corresponding sequences are provided in Table S3.

### 3.3. Intraspecific and Interspecific Variation Comparison

**Figure 4:**
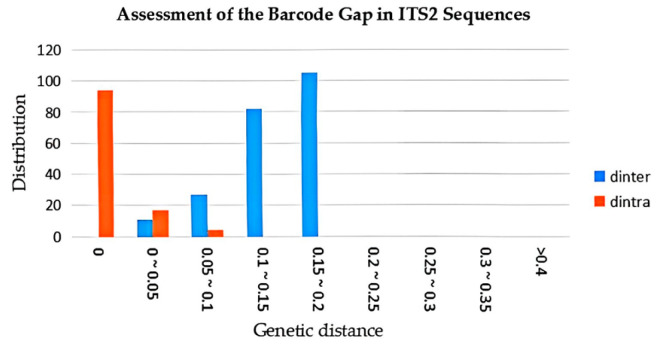
Relative distribution of interspecific divergence and intraspecific among congenetic species for ITS2.

The sheared sequences were annotated and the K2P parameter model was selected to calculate the intraspecific genetic distances (d_intra_) and interspecific genetic distances (d_inter_) using MEGA 11.0 software. The d_intra_ for three herbs and their related species ranged from 0.000 to 0.062, while the d_inter_ ranged from 0.009 to 0.220 (Figure 4, Table S4). This clear disparity comfirms that the d_inter_ far exceed the d_intra_. Furthermore, the average d_intra_ of ASR was 0.000, the average d_intra_ of the APR was 0.000, and the average d_intra_ of the LPR was 0.000. The average d_inter_ between ASR and LPR was 0.141, and the d_inter_ between S9 and ASR was 0.000. The average d_inter_ between ASR and APR was 0.136, and the average d_inter_ between APR and LPR was 0.047. The Biological Barcode Consortium proposes the comparison of the values of d_intra_ and d_inter_ to evaluate the discrimination effect of barcode sequences. An ideal barcode d_inter_ should be much larger than the d_intra_, and if the value of the d_inter_ divided by the d_intra_ is less than 1, the barcode sequence is not an ideal barcode sequence for that species. In this experiment, the d_inter_ was significantly greater than the d_intra_, and the ITS2 sequence was suitable for the differentiation of ASR, LPR, and APR.

### 3.4. ITS2 Secondary Structure Prediction and Phylogenetic Tree Construction

#### 3.4.1. ITS2 Secondary Structure Prediction

**Figure 5:**
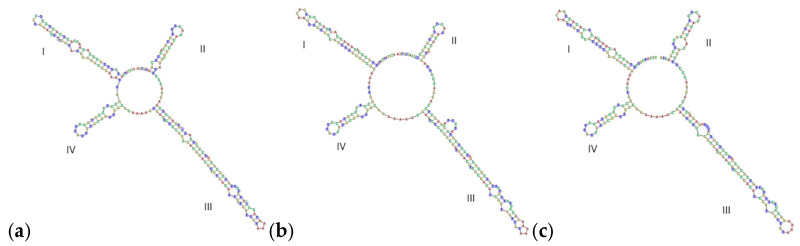
The secondary structures of internal transcribed spacer 2 region: (**a**) ASR; (**b**) APR; (**c**) LPR.

The secondary structure can also separate ASR, LPR, and APR effectively. From Figure 5, it can be seen that the ITS2 secondary structures of ASR, LPR, and APR were significant different. Helix IV is relatively conservative, suggesting that ASR, LPR, and APR all have two rings. The main differences were found in Helix I, II, and III. ASR has four rings on Helix I, two rings on Helix II, and four rings on Helix III. APR has three rings on Helix I, one ring on Helix II, and five rings on Helix III. LPR has three rings on Helix I, two rings on Helix II, and four rings on Helix III. In addition, the length and angle of Helix I, II, III, and IV are also different.

#### 3.4.2. Phylogenetic Tree Construction

**Figure 6:**
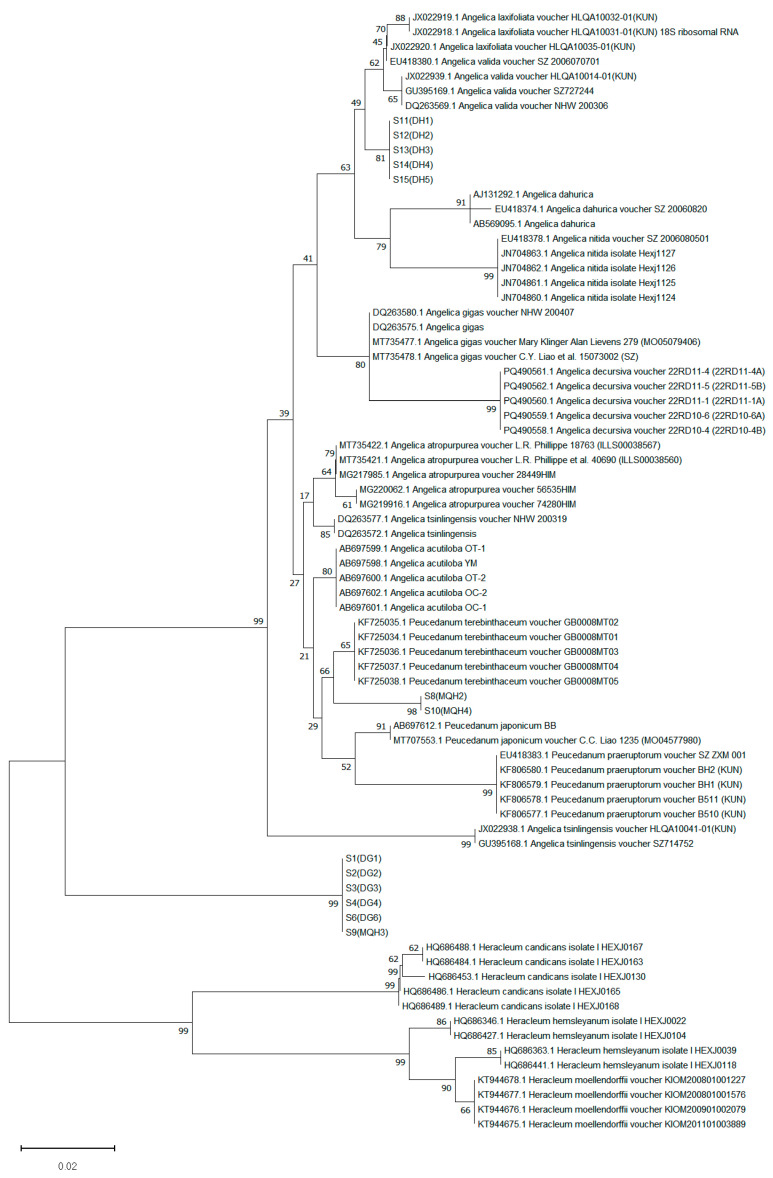
The neighbor-joining tree comprising the 13 samples and their related species.

All successfully amplified sequences from the samples along with their related species were imported into MEGA 11.0 software. A neighbor-joining (NJ) phylogenetic tree was constructed using the Kimura 2-parameter (K2P) model, with bootstrap values calculated from 1000 replicates. A bootstrap support threshold >50% was set as the criterion for successful species identification. The phylogenetic tree exhibited a deeply bifurcating topology with three strongly supported (Bootstrap = 99%) primary clades at the first divergence node (Figure 6). These three clades corresponded precisely to the species of *Heracleum*, ASR, and a combined cluster of the species of *Angelica* and *Peucedanum*. The third and most complex clade intermingled species from the genera *Angelica* and *Peucedanum*. Within this clade, LPR nested within other *Peucedanum* species, while the authentic APR clustered securely within the *Angelica* species. The ASR and the species of *Heracleum* each formed a distinct monophyletic clade. ASR, LPR, and APR were each clearly separated from other related species in the phylogenetic tree, supporting the use of phylogenetic analysis for their accurate authentication. Notably, the *Heracleum* clade comprised samples marketed as APR, which provides unambiguous evidence that the market name APR is applied to two phylogenetically distant entities: the authentic APR within the *Angelica* clade, and adulterants from the genus *Heracleum*.

## 4. Discussion

This study constructed a phylogenetic tree encompassing the genera *Angelica*, *Heracleum*, and *Peucedanum.* This approach not only confirmed the utility of the ITS sequence for discriminating ASR, APR, and LPR but also achieved precise traceability of market adulterants. Firstly, we provide the first phylogenetic confirmation of the absolute specificity of the ITS sequence in authenticating ASR. All ASR samples formed distinct genetic clusters separate from their related species, establishing a reliable molecular basis for quality control of this medicinal material. Secondly, our findings reveal systematic patterns of market substitution. Phylogenetic analysis confirmed clear genus-level adulteration in APR materials, with significant genetic divergence observed between the authentic *Angelica*-derived APR and substitutes from the genus *Heracleum*. This finding provides scientific evidence for standardizing the herbal market. Finally, this study not only ensures the accurate authentication of ASR but also provides a transferable methodological framework for identifying other Chinese medicinal materials susceptible to adulteration with related species.

However, this study has two main limitations. Firstly, the Sanger sequencing-based molecular identification method used is relatively costly, making it difficult for it to meet the demands of large-scale market screening. Secondly, the current method requires a period of 24~48 h from DNA extraction to obtaining sequencing results, which lacks the timeliness needed for rapid on-site inspection. These technical and economic constraints currently limit the practical deployment of this method in grassroots-level drug regulation departments.

## 5. Conclusions

This study successfully established an accurate identification system for ASR and related species using ITS2 sequence and secondary structure analysis. The results showed an 86.67% PCR amplification success rate, significantly greater interspecific genetic distances (0.009~0.220) than intraspecific distances (0.000~0.062), distinct evolutionary clades with bootstrap support over 60%, and diagnostic stem–loop structural variations. These findings support the development of rapid SNP-based authentication methods and the expansion of DNA barcode databases to advance quality control in traditional Chinese medicine.

## Figures and Tables

**Table 1:** Quality assessment of total DNA for samples S1–S15.

Sample	Concentration (ng/μL)	A_260_/A_280_
S1	482.8	2.07
S2	651.5	1.93
S3	480.0	2.07
S4	615.7	1.96
S5	33.0	1.97
S6	541.1	1.98
S7	19.2	1.78
S8	96.3	2.09
S9	97.8	1.97
S10	149.2	2.13
S11	260.2	2.13
S12	288.1	2.15
S13	62.3	2.12
S14	160.6	2.01
S15	249.4	2.16

**Table 2:** Properties of the internal transcribed spacer 2 region of the 13 samples.

Samples	Length (bp)	GC Content (%)	Variable Sites
			1 1 1 1 1 2 2 3 3 3 3 3 3 3 3 3 4 4 4 4 4 4 4 4 4 5 5 5 5 5 5 6 6 6 6 6 6 7 9 2 3 6 7 8 4 9 1 2 3 4 5 6 7 8 9 0 1 2 3 4 5 6 7 8 0 1 5 7 8 9 1 2 3 4 5 6
S1, S2, S3, S4, S6	229	54.15	ACTGCACCTCTCGTGGAGCTGTACTGGATGCGGAATTGGC
S8, S10	227	55.51	GCGCACACATGAGAAGTTGTGCCGTTTGGCGAACTGGCCT
S11, S12, S13, S14, S15	227	55.51	GAGCACACACTGAGAAGTTGTGCCGGTTGCGAATTGGCC
S9	227	54.62	ACTGCACCTCTCGTGGAGCTGTACTGGATGCGGAATTGGC
1 1 1 1 1 1 1 1 1 1 1 1 1 1 1 1 1 1 1 1 1 1 1 1 1 1 1 1 1 1 1 1 1 1 1 1 1 1 1 1 1 1 1 1 1 1 1 1 1 1 6 6 7 7 7 7 7 7 7 7 8 8 8 8 8 8 8 9 9 9 9 9 9 0 0 0 0 0 0 1 1 1 1 1 1 1 1 1 2 2 2 2 2 2 2 2 3 3 3 3 3 3 3 3 4 4 4 4 4 4 4 5 5 5 5 5 5 5 5 5 5 6 6 6 7 9 1 2 3 4 5 6 7 8 0 1 4 6 7 8 9 0 1 3 4 5 1 3 4 7 8 9 0 2 3 4 5 6 7 8 9 0 1 2 4 5 7 8 9 0 1 3 4 5 6 8 2 3 4 5 7 8 9 0 1 2 3 4 5 6 7 8 9 0 1 4 TCCGTGCCTGTTCGGTTGCGCATAGTCCGCGACGGACGTTGCATTGTGGTGTAATACCTCATGTCTTGTCGCGC CGTACCTTGCGCGTTGGCGAACATCCGGCACGGACGTCGACTCGGTGTTGAAGGCCTCTTGTCTTGTCGCGCG TCGTACCTTGCGCGTTGGCGAACATCCGGCATGGACGTCGACTCGGTGTTGAAAGACCTCTTGTCTTGTCGCGC TCCGTGCCTGTTCGGTTGCGCATAGTCCGCGACGGACGTTGCATTGTGGTGTAATACCTCATGTCTTGTCGCGCG 1 1 1 1 1 1 1 1 1 1 1 1 1 1 1 1 1 1 1 1 1 1 1 2 2 2 2 2 2 2 2 2 2 2 2 2 2 2 2 2 2 2 2 2 2 2 2 2 2 2 2 6 6 6 6 6 7 7 7 7 7 7 7 7 7 7 8 8 8 8 9 9 9 9 0 0 0 0 0 0 0 0 1 1 1 1 1 1 1 1 1 1 2 2 2 2 2 2 2 2 2 2 5 6 7 8 9 0 1 2 3 4 5 6 7 8 9 0 1 2 4 6 7 8 9 0 2 3 4 5 7 8 9 0 1 2 3 4 5 6 7 8 9 0 1 2 3 4 5 6 7 8 9 GAATCCGCGTCATCTTAGTGAGCTCAAGGACCTTAGGCGGCACACACTTTGTGCACTTTCAATG AATCCTCGTCATTTTAGAGAGCTCCAGGACCTTCGGCAGCACACACTCTGTGCGCTTCGACTG GAATCCTCGTCATCTTAGCGAGCTCCAGGACCTTAGGTAGCACATACTCTGTGCGCTTCGACTG AATCCGCGTCATCTTAGTGAGCTCAAGGACCTTAGGCGGCACACACTTTGTGCACTTCGAATG			

## Data Availability

The original contributions presented in this study are included in the article/Supplementary Materials. Further inquiries can be directed to the corresponding authors.

## Supplementary Materials

The following supporting information can be downloaded at: https://www.mdpi.com/article/10.3390/genes16111333/s1, Figure S1: Agarose gel electropherogram of the ITS2 fragment PCR product of Angelicae Sinensis Radix S1–S6; Figure S2: Agarose gel electropherogram of ITS2 fragment PCR products of Ligusticopsis Pubescens Radix S7–S10 and Angelicae Pubescens Radix S11–S15; Table S1: The informations of the collected samples; Table S2: GenBank accession numbers for the ITS2 sequences from related species of Angelicae Sinensis Radix, Ligusticopsis Pubescens, and Angelicae Pubescens Radix; Table S3: Sequence Data; Table S4: Matrix Output [15,16].

## Abbreviations

ITS2	Internal Transcribed Spacer 2
DNA	Deoxyribo Nucleic Acid
RNA	Ribonucleic Acid
PCR	Polymerase Chain Reaction
d_intra_	Intraspecific Distance
d_inter_	Interspecific Distance
K2P	Kimura 2-Parameter
NJ	Neighbor-Joining
NCBI	National Center for Biotechnology Information
CTAB	Cetyltrime Thylammonium Bromide
IUB	International Union of Biochemistry
