# Supplementary material for: DNA Barcoding Identification of Angelicae Sinensis Radix and Its Adulterants Based on Internal Transcribed Spacer 2 Region and Secondary Structure Prediction
Source: Genes (Basel). 2025 Nov 5;16(11):1333. doi: 10.3390/genes16111333 (PMC12652221; doi:10.3390/genes16111333)
Supplement: Supplementary file 1 [file genes-16-01333-s001.zip › Figure S1.pdf]

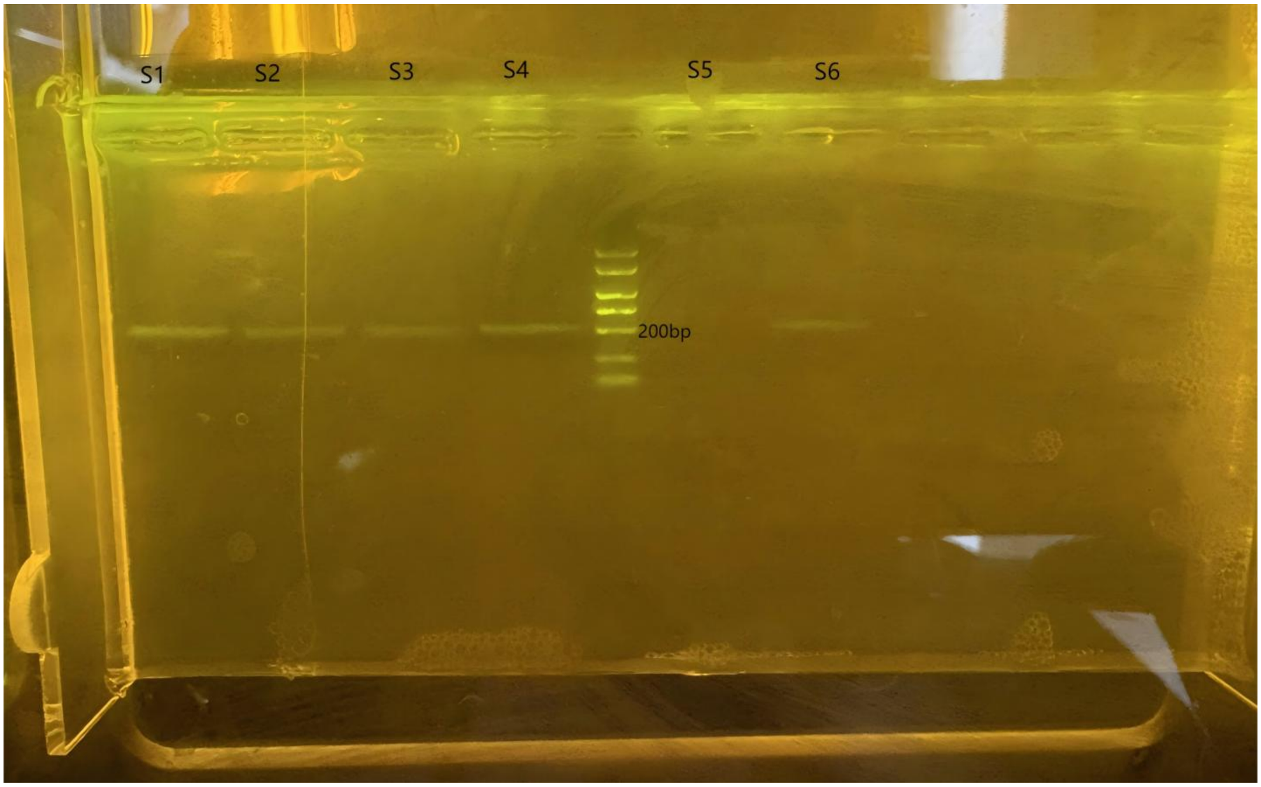

Figure S1: Agarose gel electropherogram of the ITS2 fragment PCR product of Angelicae Sinensis Radix S1-S6.

From left to right, S1, S2, S3, S4, S5, S6, a total of 6 samples, the last three wells are blank wells.
