# Supplementary material for: DNA Barcoding Identification of Angelicae Sinensis Radix and Its Adulterants Based on Internal Transcribed Spacer 2 Region and Secondary Structure Prediction
Source: Genes (Basel). 2025 Nov 5;16(11):1333. doi: 10.3390/genes16111333 (PMC12652221; doi:10.3390/genes16111333)
Supplement: Supplementary file 1 [file genes-16-01333-s001.zip › Figure S2.pdf]

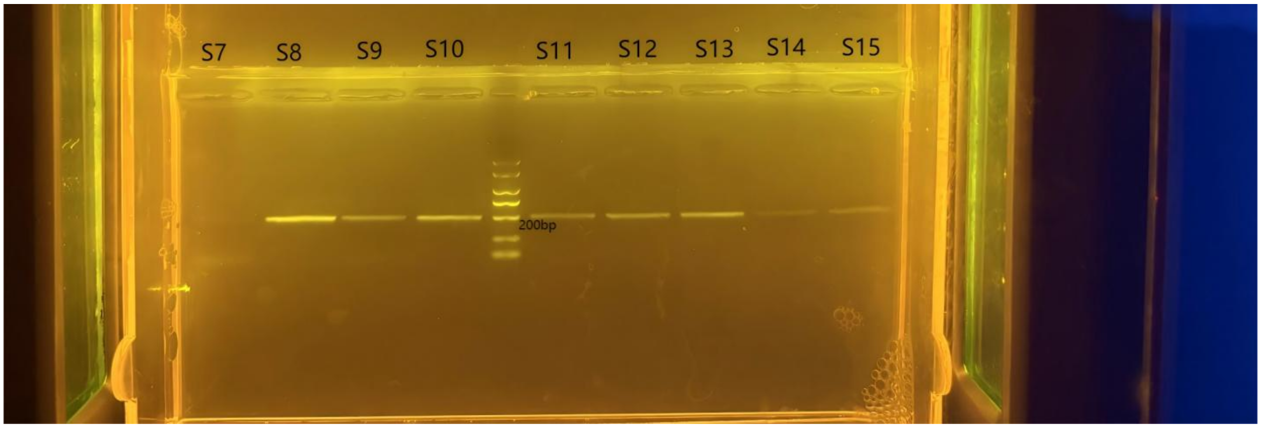

Figure S2: Agarose gel electropherogram of ITS2 fragment PCR products of Ligusticopsis Pubescens Radix S7-S10 and Angelicae Pubescens Radix S11-S15.

From left to right, S7, S8, S9, S10, S11, S12, S13, S14, S15, a total of 9 samples.
