# Supplementary material for: DNA Barcoding Identification of Angelicae Sinensis Radix and Its Adulterants Based on Internal Transcribed Spacer 2 Region and Secondary Structure Prediction
Source: Genes (Basel). 2025 Nov 5;16(11):1333. doi: 10.3390/genes16111333 (PMC12652221; doi:10.3390/genes16111333)
Supplement: Supplementary file 1 [file genes-16-01333-s001.zip › Table S1.pdf]

Table S1. The informations of the collected samples

Sample	Label	Origin
S1	Angelicae Sinensis Radix	Minxian, Gansu, China
S2	Angelicae Sinensis Radix	Minxian, Gansu, China
S3	Angelicae Sinensis Radix	Minxian, Gansu, China
S4	Angelicae Sinensis Radix	Longnan, Gansu, China
S5	Angelicae Sinensis Radix	Gansu, China
S6	Angelicae Sinensis Radix	Gansu, China
S7	Ligusticopsis Pubescens Radix	Jieyang, Guangdong,China
S8	Ligusticopsis Pubescens Radix	Huoshan, Anhui, China
S9	Ligusticopsis Pubescens Radix	Hebei, China
S10	Ligusticopsis Pubescens Radix	Shanxi, China
S11	Angelicae Pubescens Radix	Jieyang, Guangdong, China
S12	Angelicae Pubescens Radix	Jieyang, Guangdong, China
S13	Angelicae Pubescens Radix	Huoshan, Anhui, China
S14	Angelicae Pubescens Radix	Huoshan, Anhui, China
S15	Angelicae Pubescens Radix	Gansu, China
