# Supplementary material for: DNA Barcoding Identification of Angelicae Sinensis Radix and Its Adulterants Based on Internal Transcribed Spacer 2 Region and Secondary Structure Prediction
Source: Genes (Basel). 2025 Nov 5;16(11):1333. doi: 10.3390/genes16111333 (PMC12652221; doi:10.3390/genes16111333)
Supplement: Supplementary file 1 [file genes-16-01333-s001.zip › Table S2.pdf]

Table S2. GenBank accession numbers for the ITS2 sequences from related species of Angelicae Sinensis Radix, Ligusticopsis Pubescens, and Angelicae Pubescens Radix.

Species	Genera	GenBank ID	Species	Genera	GenBank ID
Heracleum	*Heracleum*	KT944676.1	*Angelica*	*Angelica*	AB697602.1
moelldorffii		KT944675.1	*acutiloba*		AB697601.1
		KT944677.1			AB697600.1
		KT944678.1			AB697599.1
Heracleum		HQ686363.1			AB697598.1
hensleyanum		HQ686441.1	*Angelica*		DQ263577.1
		HQ686346.1	*tsinlingensis*		DQ263572.1
		HQ686427.1			JX022938.1
Heracleum		HQ686489.1			GU395168.1
candicans		HQ686486.1	*Angelica*		AB569095.1
		HQ686453.1	*dahunrica*		AJ131292.1
		HQ686488.1			EU418374.1
		HQ686484.1	*Angelica valida*		EU418380.1
Angelica	*Angelica*	MG220062.1			JX022939.1
atropurpurea		MG219916.1			GU395169.1
		MG217985.1			DQ263569.1
		MT735422.1	*Peucedanum*	*Peucedanum*	KF725038.1
		MT735421.1	*terebinthaceum*		KF725037.1
Angelica		PQ490559.1			KF725036.1
decursiva		PQ490558.1			KF725035.1
		PQ490560.1			KF725034.1
		PQ490562.1	*Peucedanum*		AB697612.1
		PQ490561.1	*japonicum*		MT707553.1
Angelica gigas		MT735478.1	*Peucedanum*		EU418383.1
		MT735477.1	*praeruptorum*		KF806580.1
		DQ263575.1			KF806579.1
		DQ263580.1			KF806578.1
Angelica		JN704861.1			KF806577.1
nitida		JN704860.1			
		JN704862.1			
		JN704863.1			
		EU418378.1			
